# miR-181a/MSC-Loaded Nano-Hydroxyapatite/Collagen Accelerated Bone Defect Repair in Rats by Targeting Ferroptosis Pathway

**DOI:** 10.3390/jfb15120385

**Published:** 2024-12-20

**Authors:** Xiongjun Xu, Junming Feng, Tianze Lin, Runheng Liu, Zhuofan Chen

**Affiliations:** 1 Hospital of Stomatology, Guanghua School of Stomatology, Guangdong Provincial Key Laboratory of Stomatology, Sun Yat-sen University, Guangzhou 510055, China; xuxiongj@mail.sysu.edu.cn (X.X.); fengjm9@mail2.sysu.edu.cn (J.F.); lintz@mail2.sysu.edu.cn (T.L.); 2 The Third Affiliated Hospital of Sun Yat-Sen University, Guangzhou 510630, China

**Keywords:** mesenchymal stem cells, miR-181a, bone repair, ferroptosis, nano-hydroxyapatite scaffold

## Abstract

**Background**: The reparative regeneration of jawbone defects poses a significant challenge within the field of dentistry. Despite being the gold standard, autogenous bone materials are not without drawbacks, including a heightened risk of postoperative infections. Consequently, the development of innovative materials that can surpass the osteogenic capabilities of autologous bone has emerged as a pivotal area of research. **Methods**: Mesenchymal stem cells (MSCs), known for their multilineage differentiation potential, were isolated from human umbilical cords and transfected with miR-181a. The osteogenic differentiation of miR-181a/MSC was investigated. Then, physicochemical properties of miR-181a/MSC-loaded nano-hydroxyapatite (nHAC) scaffolds were characterized, and their efficacy and underlying mechanism in rat calvarial defect repair were explored. **Results**: miR-181a overexpression in MSCs significantly promoted osteogenic differentiation, as evidenced by increased alkaline phosphatase activity and expression of osteogenic markers. The miR-181a/MSC-loaded nHAC scaffolds exhibited favorable bioactivity and accelerated bone tissue repair and collagen secretion in vivo. Mechanistic studies reveal that miR-181a directly targeted the TP53/SLC7A11 pathway, inhibiting ferroptosis and enhancing the osteogenic capacity of MSCs. **Conclusions**: The study demonstrates that miR-181a/MSC-loaded nHAC scaffolds significantly enhance the repair of bone defects by promoting osteogenic differentiation and inhibiting ferroptosis. These findings provide novel insights into the molecular mechanisms regulating MSC osteogenesis and offer a promising therapeutic strategy for bone defect repair.

## 1. Introduction

Over the past decade, the incidence of missing teeth with bone defects among the elderly is rapidly increasing [1,2,3]. Therefore, the question of how most effectively enhance the regenerative repair of defective bone tissue represents a significant topic within the field of dentistry. Currently, the treatment of bone defects mainly relies on allogeneic bone grafting [3]. However, this treatment have many limitations, including a high susceptibility to postoperative infections. The bone repair process is tightly modulated by the dynamic balance between osteoclasts and osteoblasts. Osteoclasts mainly perform the function of bone resorption by absorbing organic matter and calcium in the bone matrix, thereby destroying the bone. In contrast, osteoblasts secrete bone matrix and induce mineralization of the bone matrix to form new bone [4,5,6].

Mesenchymal stem cells (MSCs) are a class of cells with the capacity for multidirectional differentiation potential, which is widely present in many human tissues, such as the umbilical cord, bone marrow, gingiva, and adipose tissue [7]. Furthermore, MSCs are easy to isolate and exhibit low immunogenicity [8,9]. Therefore, in terms of these advantages, MSCs have been widely used in tissue regeneration and organ injury. A number of clinical trials have demonstrated the remarkable clinical efficacy of MSCs in the treatment of a range of diseases. MSCs are currently a widely applied cell type in the studies of bone repair. MSCs have the potential for multidirectional differentiation and can be induced in vitro to differentiate into bone tissues. However, MSCs harbor the capacity for multidirectional differentiation, which limits their function in bone repair. Currently, how to effectively strengthen the pro-osteogenic effects of MSCs in bone repair remains elusive.

MicroRNAs (miRNAs) are a class of endogenous non-coding small RNAs with a length of approximately 22 nucleotides. Prior research has demonstrated that miRNAs are widely involved in a multitude of physiological processes, such as cell proliferation, cellular differentiation, and bone repair [10,11,12]. MiRNAs are transcribed by RNA polymerase II and finally transported to the RNA-induced silencing complex (RISC) [13]. The RISC binds to the double-stranded miRNAs and shears one of the strands to form mature single-stranded miRNAs. MiRNAs lack the protein-coding function, but mature miRNAs degrade the mRNAs of target genes or inhibit the translation of target genes through complete or incomplete complementary pairing with the mRNAs of the target genes, mainly in the 3’ untranslated region (UTR) [11]. The expression profiles of miRNA are significantly altered during osteogenesis. Previous studies display that miRNAs regulate osteogenic differentiation by targeting various genes involved in the osteogenic differentiation of MSCs. It has been found that miRNAs can promote osteoblast differentiation by regulating the expression of target genes [14,15,16]. For example, miR-21a was found to directly target Spry2, a key factor in osteogenic differentiation, to promote the differentiation of bone marrow MSCs to osteoblasts [17]. Moreover, both in vitro and in vivo studies supported that miR-21a accelerated new bone formation due to injury or osteoporosis [14,18]. Knockdown of miR-21a in MSCs resulted in impaired osteogenic differentiation of MSCs, poor mineralization, and reduced craniofacial bone formation in mice [18]. Recent investigations found that miR-181a promoted osteoblastic differentiation by targeting the TGF-β type I receptor (TβR-I) [15,19]. Previous studies also found that miR-27a may become involved in the process of osteogenesis by targeting GREM1 and PI3K [16,20], an important modulator of osteogenesis. However, despite the fact that the aforementioned studies have reported that these three miRNAs play a significant regulatory role in osteogenesis, there is a lack of research that directly compares the effects of these three miRNAs on osteogenic regulation. Therefore, in this study, we have selected the aforementioned miRNAs and compare their effects on osteogenic regulation. We have selected the miRNA with the most significant effect for further research.

The primary inorganic components of bone are calcium and phosphorus. A large number of investigations have shown that calcium–phosphorus compounds as bone repair materials have high biocompatibility. Hydroxyapatite (HA) is the main form of calcium–phosphorus compounds in bone tissues [21]. Both bone and teeth are mainly composed of hydroxyapatite, with about 95% of tooth enamel being composed of hydroxyapatite. Hydroxyapatite is the main inorganic component of bone tissues. It is non-toxic and has good biocompatibility. Natural bone is mainly composed of hydroxyapatite and collagen, with a highly hierarchical three-dimensional structure. Subsequent studies have shown that nanohydroxyapatite crystals attached to collagen fibers have profound effects on bone repair. Thus, nano-hydroxyapatite/collagen (nHAC) emerges as a novel bioactive material for bone repair, making nHAC a promising bioactive material for bone repair [22,23,24].

In this study, to enhance the pro-osteogenic effects of MSCs in bone repair, the MSCs with miR-181a overexpression were loaded into the nano-hydroxyapatite/collagen (nHAC). Subsequently, the physicochemical properties of miR-181a/MSC-loaded porous hydroxyapatite were then determined. The in vivo experiments demonstrated that the implantation of miR-181a/MSC-loaded hydroxyapatite enhanced the repair of the rat calvarial defects. Further studies found that miR-181a directly targeted TP53 mRNA, thus inhibiting the TP53-mediated ferroptosis pathway, leading to enhanced osteogenic capacity of MSCs. The above molecular mechanisms will provide new insights into the treatment of bone defect repair.

## 2. Methods

### 2.1. Isolation of Human Umbilical Cord MSCs

The sample collection was approved by the Institutional Review Board of the Third Affiliated Hospital of Sun Yat-sen University (RG2023-273-01). The human umbilical cord tissues were rinsed repeatedly using PBS. The blood vessels within the umbilical cord were carefully removed, and the umbilical cord tissues were cut into 1 mm^3^ sized pieces and digested in DMEM solution containing Collagenase NB4 (Sigma, St. Louis, MI, USA), Hyaluronidase (Sigma), and Neutral Protease II (Sigma) for 3 to 5 h. The cells were cultured using low-sugar DMEM medium containing 10% FBS, 100 mg/L penicillin, and 100 mg/L streptomycin. The cells were inoculated into culture flasks at a density of approximately 1 × 10^3^ cells/cm^2^, and then the flasks were placed in an incubator at 37 °C with 5% CO_2_. After 96 h, half of the culture medium was replaced, and then half of the culture medium was replaced every other day. Once the MSCs had reached 80% confluence, the cells were passaged.

### 2.2. miRNA Transfection

Based on the literature review of the introduction, three miRNAs were selected as initial screening targets, namely miR-181a, miR-21a, and miR-27a. These three miRNA mimics were synthesized by the Sangon Company. The three miRNA mimics (miR-181a, miR-21a, and miR-27a) and corresponding negative controls were transfected by using transfection reagent Lipofectamine 2000 (Thermo Fisher Scientific, Waltham, MA, USA). The final concentrations of three miRNA mimics (miR-181a, miR-21a, and miR-27a) and corresponding negative controls were 50 nM. Based on the research findings of this section, have selected choose miR-181a as the subject of further study.

### 2.3. Preparation of miR-181a-MSCs-nHAC Samples

The nano-hydroxyapatite/collagen (nHAC) was purchased from the Tianjin Sannie Bioengineering TECHNOLOGY Company (Tianjin, China) with trade name Bone^3^. MSC cells were seeded into six-well plates at a density of 1 × 10^6^ cells/mL and cultured in serum-free medium overnight. The miR-181a mimics (50 nM) were transfected into MSCs using Lipofectamine 2000 (Thermo Fisher Scientific). MSCs transfected with miR-181a at a density of 1 × 10^6^ cells/mL were dropped into porous nHAC collagen blocks until the collagen blocks were saturated.

### 2.4. Physical Phase Analysis

A number of nHAC and miR-181a-MSCs-nHAC samples were randomly selected (approximately 1 mm thickness) and uniformly pressed into thin slices. The samples were analyzed for physical phase changes using an X-ray powder diffractometer (XRD; D/max 2550 V, Rigaku, Tokyo, Japan). The test conditions were CuKα target emission source (λ = 1.5418 Å), voltage of 40 kV, current of 40 mA, scanning 2theta range of 10°–80°, step size of 0.016°, and dwell time 20 s. The data were analyzed using JCPDS (Joint Committee on Powder Diffraction Standards) and MDI JADE 6.5 software.

### 2.5. Elemental and Functional Group Analysis

Several randomly selected nHAC and miR-181a-MSCs-nHAC samples (approximately 1 mm thickness) were uniformly pressed into thin slices and analyzed for the composition of the functional groups of the samples using a Fourier Transform Infrared Spectrometer (FTIR; VERTEX 70v, Bruker Optik, Ettlingen, Germany) in total reflection mode with a resolution of 1 cm^−1^. The scanning range was 400–4000 cm^−1^. Auxiliary compositional analysis was also carried out using a laser Raman spectrometer (Raman; LabRAM HR, HORIBA Jobin Yvon, Palaiseau, France) with an emitter laser source of Nd:YAG (532.8 nm) with a scanning range of 400–4000 cm^−1^.

### 2.6. Morphological Analysis

The surface morphology of the samples was observed using a field emission scanning electron microscope (FESEM; Ultra 55, Zeiss, Oberkochen, Germany). The samples of nHAC and miR-181a-MSCs-nHAC were fixed on an electron microscope stage after 24 h of freeze-drying and sprayed with gold to make the surface conductive. The samples were operated with an accelerating voltage of 5 kV, and the elemental composition and content of the microregions of the samples were analyzed using an X-ray energy spectrometer (EDX, Cambridge, MA, USA) attached to the electron microscope.

### 2.7. Animal Study

This study was approved by the Institutional Animal Care and Use Committee of Sun Yat-sen University (SYSU-IACUC-2023-001486). The male Sprague–Dawley (SD) rats, aged 6–8 weeks, were randomly assigned to four groups. After anesthesia, the rat skin was prepared by routine hair removal and disinfection. A 2 cm long incision was made along the middle of the supraorbital brow of SD rats to reach the bone surface. The mucoperiosteal flap was separated, and a slow-speed handpiece with a 5.0 mm diameter ring bone extractor drill was used to prepare the bone defect with approximately 1 mm thickness on the left and right sides under the cooling of 4 °C saline rinsing. The bone fragments were carefully removed, and the shape of the cranial wound was trimmed. The hemostasis was stopped by compression until the view of the operative area was clear. The nano-hydroxyapatite/collagen scaffold was filled into the bone defect, and the bilateral subcutaneous fascia was carefully pulled together and sutured to cover and fix the bone grafting material. Then, the subcutaneous and skin layers were closed by layered sutures. Postoperatively, gentamycin was applied to the wounds. Penicillin sodium was injected into the muscle for 3 days to prevent infection. The animals were killed on days 28 and 56, and the samples were taken for the subsequent experiments. NC means negative control. Negative control miRNAs are designed to have no known target in the human cells. Blank means that this group did not receive any treatment.

### 2.8. Histology Analysis

The tissues were fixed in 4% paraformaldehyde and subsequently embedded in paraffin. The tissue sections were subjected to H&E staining. The tissue sections were dewaxed by xylene, then hydrated with gradient ethanol and rinsed with distilled water. The tissue sections were immersed in EDTA buffer. The tissues were stained by hematoxylin and eosin. The results of staining were observed under the microscope. Masson’s trichrome staining kit was purchased from Yike Company. Goldner’s trichrome staining kit was purchased from LEAGENE Company (Guangzhou, China). Masson’s trichrome staining and Goldner’s trichrome staining were performed according to the manufacturer’s instructions. Immunohistochemistry for Osteopontin was conducted with an Osteopontin rabbit polyclonal antibody (0806-6, HUABIO, Hangzhou, China). The cellular location of Osteopontin protein was visualized by using diaminobenzidine (DAB) reagents (ZLI-9017, Beijing Zhongshanjinqiao-BIO, Beijing, China).

### 2.9. RNA Sequencing

RNA Sequencing service was provided by Aksomics Company (Shanghai, China). In brief, the mRNA from the extracted total RNA samples was enriched by oligo (dT) magnetic beads after agarose electrophoresis and quality control. The RNA sequencing libraries were prepared by the kits, which included RNA fragmentation and reverse transcription with random primers to generate first-strand cDNA. The addition of A at the end of the double-stranded cDNA to connect to the Illumina adaptor. The cDNA library was amplified by PCR. The constructed libraries were quality checked by Agilent 2100, quantified by qPCR, and sequenced by Illumina NovaSeq 6000 sequencer (Illumina, San Diego, CA, USA).

### 2.10. qRT-PCR

Total RNA was isolated by TRIZOL Reagent (Beyotime, Nantong, China) following the manufacturer’s instructions. After extraction, the RNA samples were reversely transcribed by 1st Strand cDNA Synthesis Kit (Vazyme, Nanjing, China). The relative expression profiles of target genes were analyzed by 2^−ΔΔCT^ method.

### 2.11. Osteoblast Differentiation

Once the MSCs had reached 90–100% confluence, the osteoblast differentiation was initiated. To induce the osteoblast differentiation of MSCs, the culture medium was supplemented with 0.1 μM dexamethasone (Selleck, Houston, TX, USA), 50 μM ascorbic acid 2-phosphate (Selleck) and 10 mM glycerol 2-phosphate (Sigma-Aldrich). The culture medium was changed every three days until the indicated time points.

### 2.12. CCK8 Assay

The cell proliferation was determined by the cell counting kit-8 (CCK8, C6005, New Cell & Molecular Biotech Co., Ltd., Newcastle upon Tyne, UK). The cell suspension (100 μL/well) was inoculated in a 96-well plate. The plate was placed in an incubator to pre-culture for a period of time. Then, 10 μL of CCK-8 solution was added to each well and incubated for 2 h. The absorbance at 450 nm was determined by a plate reader.

### 2.13. Detection of Reactive Oxygen Species (ROS)

Reactive oxygen species assay kit (KGA7308, Keygen Biotech, Nanjing, China) is a kit for the detection of reactive oxygen species using the fluorescent probe DCFH-DA. Diluted DCFH-DA loading working solution was added to the cells and gently mixed. The cells were incubated at 37 °C for 30 min. After incubation, the cells were centrifuged at 1000 rpm for 3 min to remove excess DCFH-DA working solution. After washing was completed, the cells were ready for flow cytometry. ROS levels were detected on the flow cytometer using an excitation wavelength of 488 nm and an emission wavelength of 525 nm. The results were analyzed using Flowjo software 10.8.1.

### 2.14. Detection of Cell Apoptosis

Annexin V-FITC/PI kit (KGA1102, Keygen Biotech) is a kit for the detection of cell apoptosis. Cells were collected by washing twice with PBS. Then, 500 μL Binding Buffer was added to the collected cells. Then, 5 μL Annexin V-FITC dye and 5 μL Propidium Iodide dye were added. The mixed cell suspension was incubated at room temperature for 10 min. Then flow cytometry was performed for detection of apoptosis. The results were analyzed by Flowjo software 10.8.1.

### 2.15. Statistical Analysis

The experimental data were expressed as mean ± standard deviation. One-way analysis of variance (ANOVA) was employed to test the differences between multiple groups, and if the variance was homogeneous, the differences were considered significant, and multiple *t*-tests were used to analyze the data between two groups. The differences were considered statistically significant when *p* < 0.05.

## 3. Results

### 3.1. miR-181a Promoted Osteogenesis

**Figure 1:**
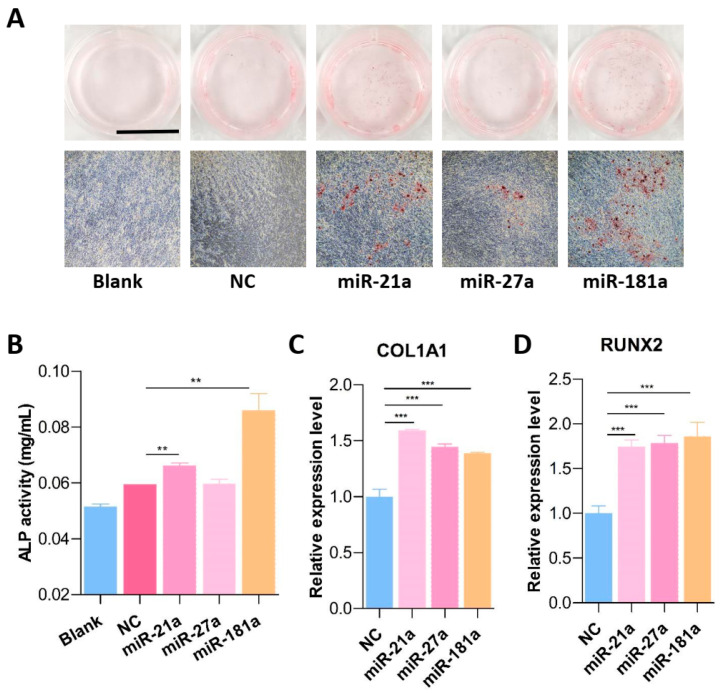
MiR-181a promoted osteogenic differentiation of human umbilical cord MSCs. (**A**) After transfection with three candidate miRNAs (miR-21a, miR-27a, and miR-181a), Alizarin red staining was used to visualize the calcium nodule granules at Day 14. The blank group did not receive osteogenic induction cocktail treatment. Scale bar: 1 cm. Magnification fold of the microscopic image: 200 fold. (**B**) Detection of ALP activity after 14 days of osteogenic induction of MSCs after transfection with three indicated miRNAs. (**C**,**D**) RT-PCR assay was used to examine the expression levels of COL1A1 (**C**) and RUNX2 (**D**) after transfection with three indicated miRNAs. (**, *p* < 0.01; ***, *p* < 0.001).

The current study isolated the human umbilical cord MSCs and validated the identity of MSCs by cell surface markers like CD105, CD73, CD90, CD44, CD166, and CD29 (Supplementary Figure S1). According to the previous investigations, we selected three candidate miRNAs (miR-21a, miR-27a, and miR-181a) that have been reported to have the function of promoting osteogenic differentiation of mouse or human mesenchymal stem cells [15,16,17]. Our results found that miR-21a and miR-181a could promote osteogenic differentiation of MSCs, both of which showed obvious staining of calcium nodule granules and strong pro-mineralization ability (Figure 1A). Subsequently, ALP activity was assessed after 14 days of osteogenic induction, and it was found that miR-181a-transfected MSCs exhibited the highest ALP activity (Figure 1B). Consistently, two important osteogenic markers, COL1A1 and RUNX2, displayed increased expression after transfection with miR-21a and miR-181a (Figure 1C,D).

### 3.2. Functional Group and Physical Phase Analysis

**Figure 2:**
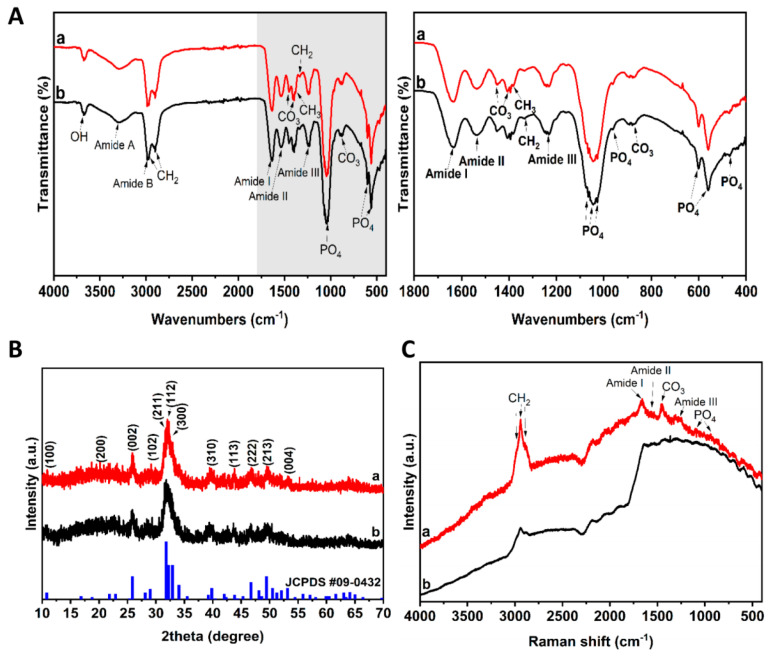
Physical phase and functional group analysis of miR-181a-MSCs-loaded nHAC scaffold. (**A**) Fourier-transform infrared spectroscopy of miR-181a-MSCs-nHAC (a) and nHAC (b). The right panel is a partial enlargement of the left panel. (**B**) X-ray diffraction patterns of the materials miR-181a-MSCs-nHAC (a) and nHAC (b). (**C**) Raman spectra of miR-181a-MSCs-nHAC (a) and nHAC (b).

Subsequently, the FTIR spectra of nHAC and miR-181a-MSC-nHAC samples were examined (Figure 2A). Combined with Supplementary Table S1, it can be seen that the absorption bands at 462, 472, 561, 575, 601, 962, 1026, 1044, 1066, and 1092 cm^−1^ are the characteristic absorption bands of PO4^3−^ within the structure of hydroxyapatite, whereas the absorption bands at 871, 879, 1408, 1450, and 1558 cm^−1^ belong to the characteristic absorption bands of AB-type carbonate, indicating that the sample contains AB-type carbonated hydroxyapatite and carbonate partially replaces the phosphate group and hydroxyl group in hydroxyapatite. According to the amide III band (1231, 1240 and 1250 cm^−1^), amide II band (1535 cm^−1^), amide I band (1634 cm^−1^), amide B band (2990 cm^−1^), amide A band (3298 cm^−1^), and the characteristic peaks of C-H (1335, 1381, 2899 and 2969 cm^−1^), we found that the samples contain collagen. The analysis shows that the sample contains collagen composition with typical three-stranded helical structural features and it indicates that the sample retains the characteristic structure of natural collagen. The strong and wide absorption band at 3673 cm^−1^ is the O-H stretching vibration absorption band, which is caused by the absorption of water vapor in the air. In addition, the nHAC complex with miR-181a-MSC did not significantly change the composition and functional group composition of the samples, indicating that it still retained good bioactive components. According to the results of XRD patterns (Figure 2B), based on the major XRD diffraction peaks and their intensities and half-peak widths, the main phase composition of both samples is weakly crystallized hydroxyapatite. The molecular formula Ca_10_(PO_4_)_6_(OH)_2_ is similar to the stoichiometric hydroxyapatite standard diffraction spectral card No. JCPDS PDF#09-0432. The characteristic peaks of other phases were not detected. In addition, according to the analysis of the diffraction peaks near the crystal surface of (002), the sample contains a certain amount of amorphous material. There is no significant difference in the diffraction peaks between the samples in the two groups, indicating that the loading of the miR-181a-MSC did not affect the main physical phase composition of the material, and the material can still maintain a highly active weakly crystallized state.

The Raman analysis further confirmed the above results (Figure 2C), and the maps presented typical information on collagen and carbonate apatite. Interestingly, in the miR-181a-MSC-nHAC sample, the Raman vibrational bands of collagen were significantly enhanced compared to the nHAC sample. We speculated that MSCs transfected with miR-181a induced the degradation of apatite minerals and facilitated the secretion of collagen, which were both conducive to the enhancement of the bioactivity of the samples. These findings may shed light on the modulation of the calcium–phosphate inorganic order mineralization in collagen.

### 3.3. Morphological Analysis

**Figure 3:**
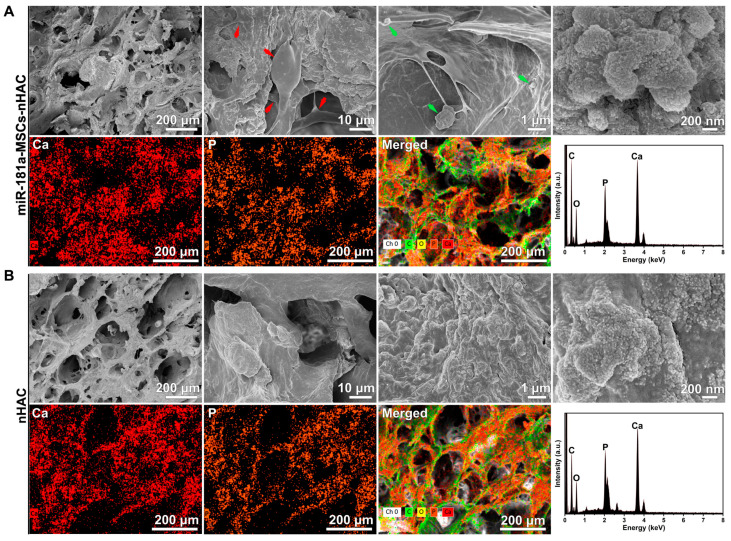
SEM photographs and EDX emission energy spectra of the surfaces of indicated biomaterials. (**A**) SEM photographs and EDX emission energy spectra of the surfaces of miR-181a-MSCs-nHAC. (**B**) SEM photographs and EDX emission energy spectra of the surfaces of nHAC. The miR-181a-MSCs were indicated by the red arrows, and clustered nanorods were shown by the green arrows.

According to the morphological analysis, the samples were all in a connected porous structure, and the nanoscale amorphous particles were uniformly distributed in the colloidal matrix (Figure 3). The state of the cells can be clearly observed in the merged image. The miR-181a-MSCs adhered well and spread on the surface of the material, which is indicated by the red arrows in the figure. Some miR-181-MSCs adhered to the surface of the material, and the miR-181a-MSCs migrated and grew into the voids of the material. The miR-181a-MSCs stretched out the pseudopods to cross-link with the surrounding cells. The miR-181a-MSCs displayed active cell metabolism, which suggests that miR-181a-MSC-loaded nHAC have excellent potential for bone defect repair.

### 3.4. miR-181a/MSC-Loaded nHAC Significantly Enhanced the Repair of Rat Calvarial Defects

**Figure 4:**
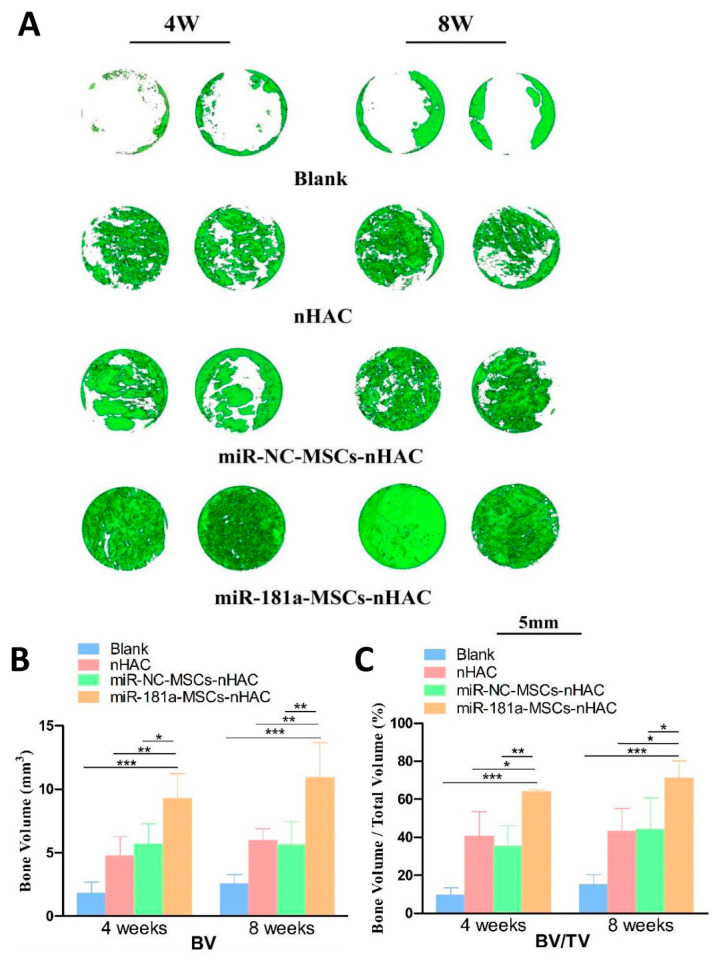
The miR-181a/MSC-loaded nHAC composites significantly enhanced the repair of rat calvarial defects. (**A**) The micro-CT assay showed 3D reconstruction of cranial defects in rats. (**B**) The bone volume was calculated at 4 weeks and 8 weeks. (**C**) The BV/TV ratio was calculated at 4 weeks and 8 weeks. (*, *p* < 0.05; **, *p* < 0.01; ***, *p* < 0.001).

One month after surgery, the 3D reconstruction images of the cranial bone defect were obtained. In the blank group at 8 weeks (Figure 4A), the bone wall edges were larger than those in the blank group at 4 weeks. However, the central area was empty, and the continuity of the bone defect was still not restored. nHAC group and miR-NC-MSCs-nHAC group had the edge of the neonatal bone tissues crawling to the central area of the defect, and the defect area became smaller. The new bone density and trabecular thickness of the implanted area increased and the bone marrow cavity decreased compared with that at 4 weeks. The defects of the miR-181a-MSCs-nHAC group were completely filled with material and new bone, which suggests that the degree of mineralization of the new bone increased, and the bone tissues became more mature. Three-dimensional morphometric analyses showed that the newborn bone volume fraction (bone volume, BV) in the miR-181a-MSCs-nHAC group was significantly higher than that in the blank group, the nHAC group, and the miR-NC-MSCs-nHAC group (Figure 4B). The miR-181a-MSCs-nHAC group’s bone volume fraction (BV/TV) was significantly higher than that of the blank, nHAC, and miR-NC-MSCs-nHAC groups (Figure 4C), since larger values of BV/TV and BV are associated with a more mature and stable bone structure.

### 3.5. miR-181a/MSC-Loaded nHAC Accelerated the Bone Tissue Repair and Collagen Secretion

**Figure 5:**
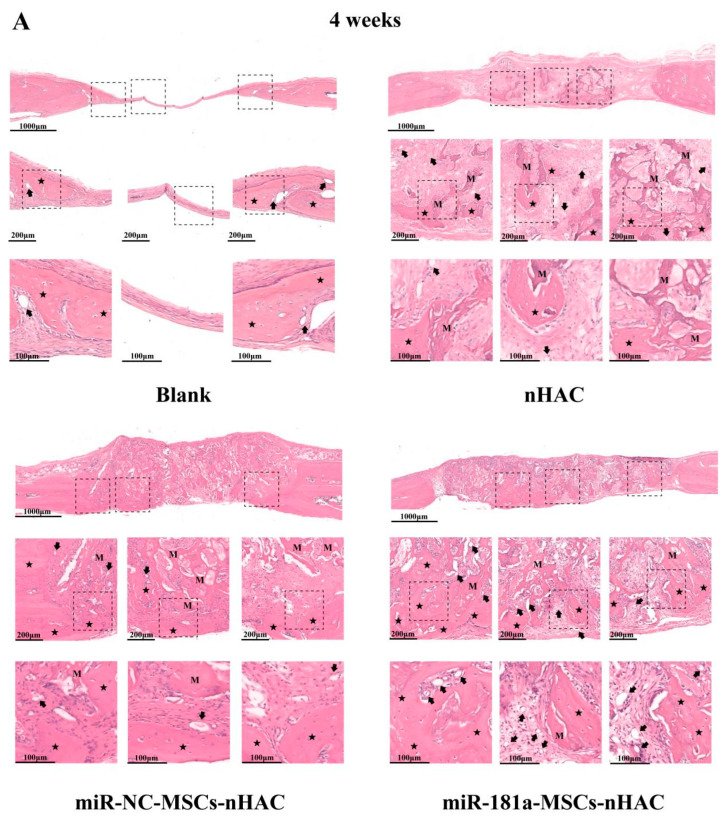

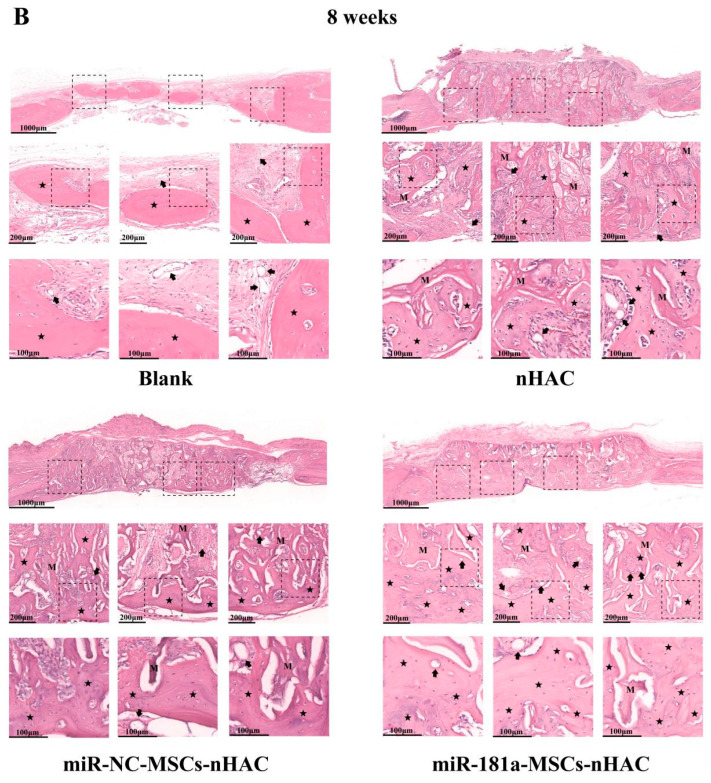
(**A**) The miR-181a/MSC-loaded nHAC composites accelerated the bone tissue repair. The H&E staining was performed at 4 weeks post-surgery in rats. Black star indicates newly formed bone. Letter M indicates collagen fiber. Arrow indicates microvessel. (**B**) The miR-181a/MSC-loaded nHAC composites accelerated the bone tissue repair. The H&E staining was performed at 8 weeks post-surgery in rats. Black star indicates newly formed bone. Letter M indicates collagen fiber. Arrow indicates microvessel.

Subsequently, histological staining was conducted using both H&E and Masson’s trichrome techniques to facilitate observation of the morphological alterations. In the blank group, we could only see a small amount of new bone growth at the edge of the defects without any biomaterials. In the nHAC group, a hydroxyapatite and collagen bone cubing material was placed in the defect to provide a scaffold for the osteoblasts, and therefore, a little new bone and blood vessels were attached to the material in the center of the defect (Figure 5A,B). However, the biological activity of the bone cube material is low due to the lack of osteoblasts. In the miR-NC-MSCs-nHAC group, we added human miR-NC-MSCs to the nHAC scaffold but failed to significantly improve the osteogenic effect. This is because MSCs have the biological characteristic of maintaining their own stemness. For instance, they need to receive specific biological signals to be activated and then differentiate to exert their regenerative ability to repair bone defects. According to the in vitro experiments, miR-181a has been shown to activate MSCs and promote their osteogenic differentiation. Therefore, we added MSCs stimulated by miR-181a to the nHAC scaffold in the miR-181a-MSCs-nHAC group and observed that a large number of new bone growths were observed both at the edges and the center of the defects, and neovascularization could be seen around the new bone (Figure 5A,B), indicating that miR-181a can effectively stimulate the osteogenic differentiation of MSCs. In addition, we also observed the cytotoxicity on other organs, and no significant morphological changes were found in most of the vital organs (Supplementary Figure S2).

**Figure 6:**
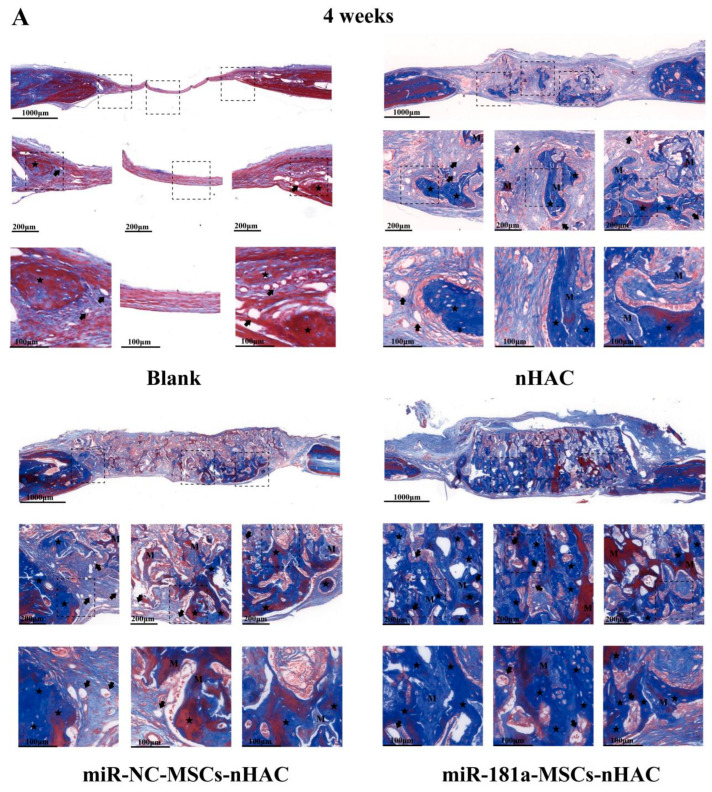

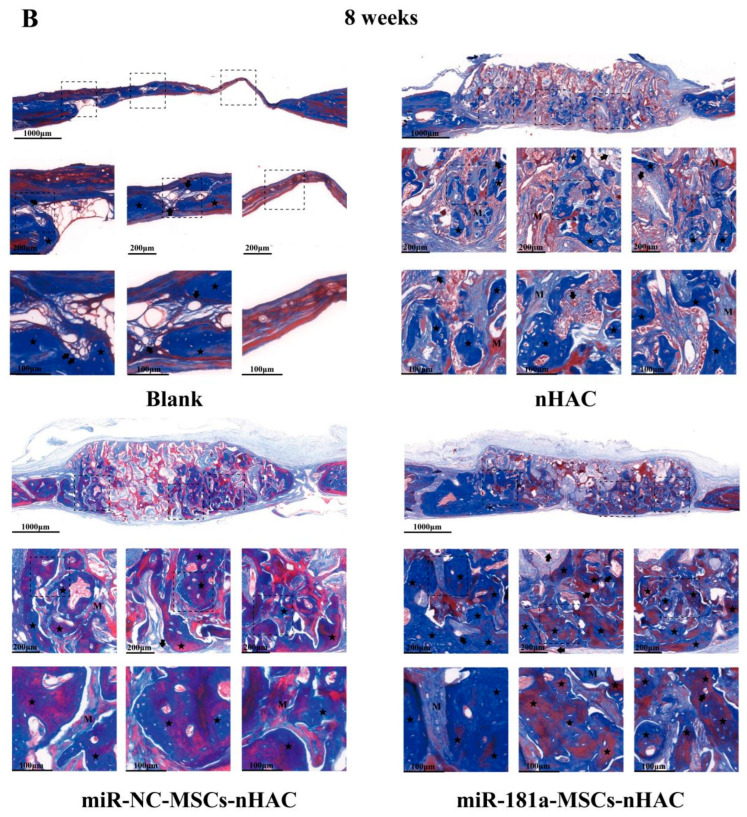
(**A**) The miR-181a/MSC-loaded nHAC composites accelerated collagen secretion. The Masson staining was performed at 4 weeks post-surgery in rats. Black star indicates newly formed bone. Letter M indicates collagen fiber. Arrow indicates microvessel. (**B**) The miR-181a/MSC-loaded nHAC composites accelerated collagen secretion. The Masson staining was performed at 8 weeks post-surgery in rats. Black star indicates newly formed bone. Letter M indicates collagen fiber. Arrow indicates microvessel.

**Figure 7:**
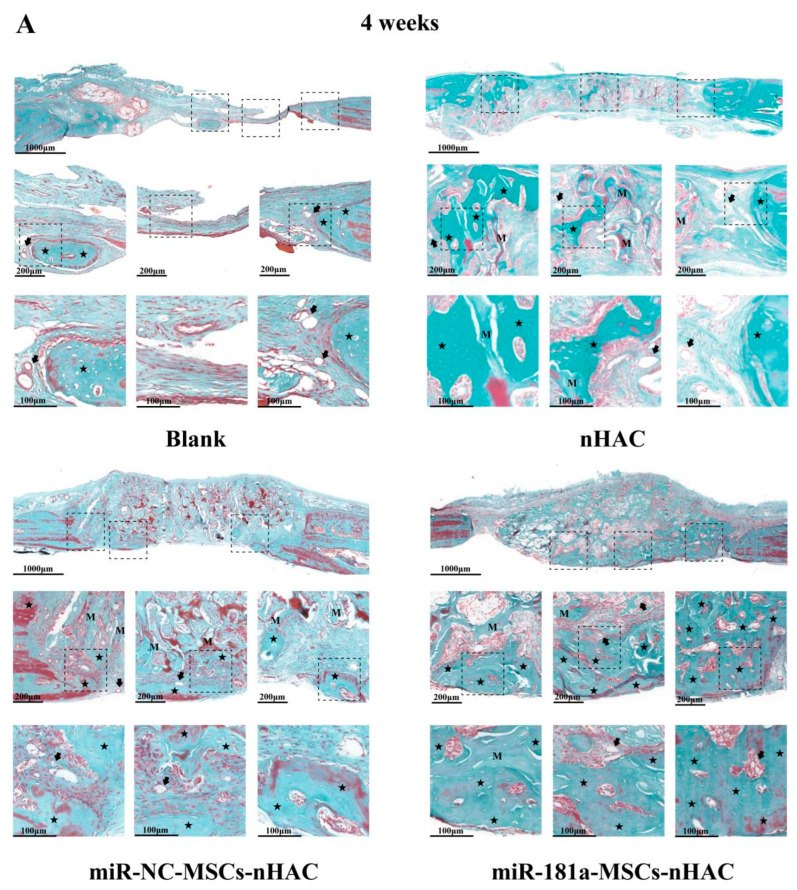

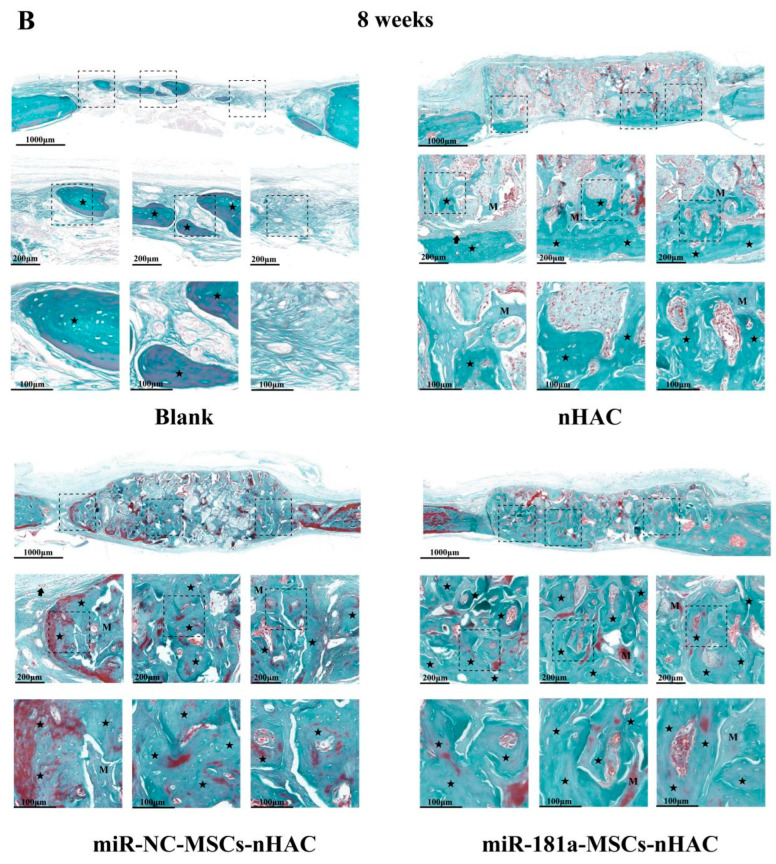
(**A**) The miR-181a/MSC-loaded nHAC composites accelerated bone regeneration. The Goldner’s trichrome staining was performed at 4 weeks post-surgery in rats. Black star indicates newly formed bone. Letter M indicates collagen fiber. Arrow indicates microvessel. (**B**) The miR-181a/MSC-loaded nHAC composites accelerated bone regeneration. The Goldner’s trichrome staining was performed at 8 weeks post-surgery in rats. Black star indicates newly formed bone. Letter M indicates collagen fiber. Arrow indicates microvessel.

It was observed that in the miR-181a-MSCs-nHAC group, the newborn bone was connected in patches and almost filled the bone defect area, and most of the newborn bone tissues were reddish stained in the center, suggesting that the newborn bone tissues had basically matured (Figure 6A,B). According to the results of Goldner’s trichrome staining, similar results were found, and the miR-181a-MSCs-nHAC group exhibited the most significant bone regeneration (Figure 7A,B). Moreover, we also performed immunohistochemical (IHC) staining of Osteopontin (OPN) in the rat tissues at 4 weeks (Supplementary Figure S3). Osteopontin is a multifunctional protein and is considered to play an important role in bone remodeling and biomineralization. According to the IHC results, overexpression of miR-181a significantly enhanced the expression of Osteopontin. These results indicate that miR-181a can effectively stimulate mesenchymal stem cell osteogenic differentiation, angiogenesis, and collagen secretion to promote the repair of bone defects.

### 3.6. miR-181a Targeted TP53/SLC7A11 Signaling Pathway

**Figure 8:**
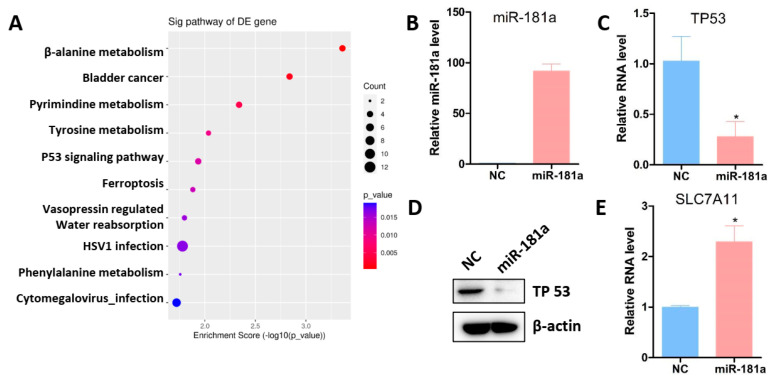
The miR-181a targeted TP53-mediated ferroptosis signaling pathway. (**A**) After transfection with miR-181a in MSCs, RNA-seq data revealed that miR-181a down-regulated various signaling pathways, including ferroptosis signaling pathway. (**B**) RT-PCR was performed to detect the transfection efficiency of miR-181a in MSCs. (**C**) Overexpression of miR-181a reduced the expression of TP53. (**D**) Overexpression of miR-181a reduced the protein level of TP53. (**E**) RT-PCR was performed to detect the expression of SLC7A11 after transfection of miR-181a. (*, *p* < 0.05).

To further explore the molecular mechanism of miR-181a in promoting osteogenic differentiation of MSCs, we conducted RNA-seq following miR-181a transfection. The RNA-seq data revealed that multiple signaling pathways were repressed by miR-181a, such as the TP53 signaling pathway and ferroptosis signaling pathway (Figure 8A). Through bioinformatic prediction, we found that TP53 might be a direct target of miR-181a (Figure 8A). We subsequently found that ectopic expression of miR-181a significantly reduced TP53 expression (Figure 8B–D). Previous investigations showed that TP53 induced ferroptosis in tumor cells by repressing the transcription of SLC7A11 and SLC7A11 protected cells from oxidative stress damage and lipid peroxidation. Furthermore, our findings indicate that miR-181a can recover the expression of SLC7A11 (Figure 8E), leading to the alleviation of ferroptosis.

### 3.7. Overexpression of miR-181a Suppressed Ferroptosis

**Figure 9:**
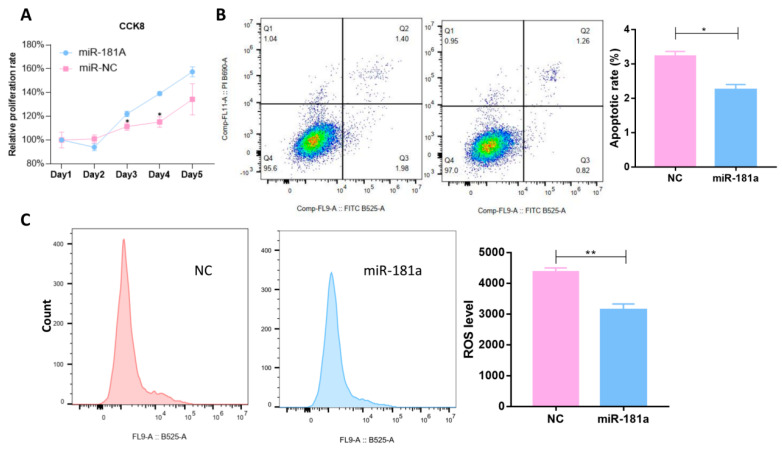
The miR-181a promoted cell proliferation by inhibiting ROS production. (**A**) After transfection with miR-181a in MSCs, CCK8 assay revealed that miR-181a enhanced cell proliferation. (**B**) Flow cytometry assay was performed to detect the cell apoptosis after overexpression of miR-181a in MSCs. (**C**) Flow cytometry assay showed that overexpression of miR-181a reduced ROS production. (*, *p* < 0.05; **, *p* < 0.01).

Ferroptosis is an iron-dependent, non-apoptotic form of programmed cell death. It is characterized by two main biochemical characteristics, namely iron accumulation and lipid peroxidation. In that context, ferroptosis is a ROS-dependent form of cell death. We performed CCK8 assay, apoptosis assay, and ROS detection assay to monitor the effect of miR-181a on ferroptosis. According to the results, we found that overexpression of miR-181a significantly promoted cell proliferation (Figure 9A) and inhibited cell apoptosis (Figure 9B). Moreover, overexpression of miR-181a also suppressed ROS production (Figure 9C). These results indicated that miR-181a may suppress ferroptosis and, thus, promote cell proliferation.

**Figure 10:**
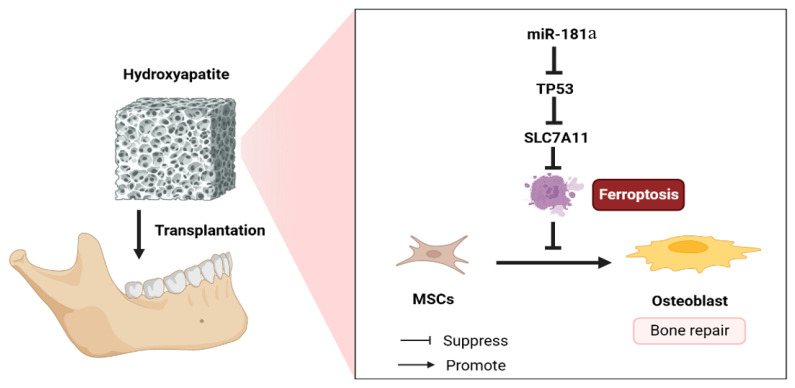
The hypothetical model. The miR-181a/MSC-loaded nano-hydroxyapatite/collagen accelerated bone defect repair by targeting TP53-mediated ferroptosis signaling pathway.

In conclusion, the results of our experiments demonstrated that the implantation of miR-181a/MSC-loaded porous nHAC significantly enhances the rat calvarial defects by inhibiting the TP53/SLC7A11-mediated ferroptosis pathway (Figure 10).

## 4. Discussion

The findings of this study indicate that the use of miR-181a/MSC-loaded porous hydroxyapatite can significantly enhance the osteogenic differentiation of mesenchymal stem cells. Subsequent mechanistic studies found that miR-181a directly targeted the TP53/SLC7A11 pathway and ultimately inhibited the ferroptosis, leading to the enhancement of bone repair. The present study has identified a novel molecular mechanism through which miRNA regulates the osteogenic differentiation of MSCs, thereby providing a new strategy for the promotion of bone defect repair.

Periodontal diseases and jaw bone defects resulting from oral and maxillofacial malignancies or trauma are frequently accompanied by tooth loss, which represents a significant health risk. Bone grafts for dental implants are derived from a variety of sources, including autogenous, allogenous, and xenograft bone. Compared with autogenous bone grafts, bovine hydroxyapatite (BHA) has the advantage of being more widely available and easy to apply [21,25]. Furthermore, patients do not require additional surgical intervention to obtain bone grafts. Compared with the chemically synthetic hydroxyapatite (SHA), SHA is produced by physically sintering or chemically treating to remove the organic components, which can retain the loose and connected pore structure of natural bone tissues during the preparation process. The degradation and resorption of BHA in the body is fast, which is conducive to the growth of the new bone into the area of the bone defects. BHA has better osteoconductive properties. Its reliable osteogenic effect has been widely proven in clinical practice. However, the source of pig is more extensive than that of cattle. This raised a question about whether hydroxyapatite from pig bone has similar or superior osteogenic efficacy to that of bovine bone. Some studies have pointed out that compared with bovine bone, porcine bone, and human bone have more similar natural pore structure and composition and have favorable results in small-scale clinical applications. Consequently, porcine hydroxyapatite (PHA) may represent a novel source for dental implants.

The results demonstrated that a considerable number of MSCs were attached to the surface of the material. In comparison to the nHAC samples, the surface microstructure of the miR-181a-MSCs-nHAC samples exhibited a sparser configuration, with greater exposure to the nanohydroxyapatite particles. The clustered nanorods were visible (shown by the green arrows in Figure 3), which were similar to the micro- and nanostructures formed by the mineralization of calcium–phosphorus inorganic compounds. EDX assay showed that the calcium–phosphorus molar ratios were similar in all the samples (around 1.88), but the calcium–phosphorus content of miR-181a-MSCs-nHAC samples was significantly lower than that of nHAC samples, which confirmed the IR and Raman results. These results suggested that the mineral content in miR-181a-MSCs-nHAC samples was altered, and the cellular activity may have accelerated the dissolution of nanoparticles, exposing the collagen matrix and consequently enhancing the cellular activity. These effects synergistically promoted collagen secretion and elevated overall collagen content. It is proposed that miR-181a-MSCs may enhance the biological activity of the samples, thereby facilitating the formation of bone matrix and the bone repair process.

Iron is a common and essential metal element in the human body, and iron homeostasis plays an extremely important role in a series of biological activities such as cell growth, cell division, and respiration. It has been found that abnormal iron deposition in the tissues of the body causes cell and tissue damage. Clinical studies have shown that bone loss is common in patients with iron overload-related diseases. Experimental studies have found that abnormal iron overload impairs the osteogenic capacity of osteoblasts while inhibiting the differentiation of stem cells to osteocytes, ultimately leading to a significant reduction in bone mass [26]. These studies indicate that iron metabolic homeostasis plays a significant role in osteogenic differentiation. However, the precise manner by which iron metabolism regulates osteoblast differentiation during the process of bone differentiation and, thus, influences the development of osteoporosis remains unclear.

It was demonstrated that TP53-induced ferroptosis in tumor cells is mediated by the repression of SLC7A11 transcription [27]. SLC7A11 is a member of the solute carrier family and belongs to the cystine/glutamate reverse transporter proteins, which are involved in amino acid transport across the cytoplasmic membrane of the cell. The SLC7A11 gene is located in the region of the human chromosome 4q28-q32 and encodes the amino acid transporter carrier xCT. SLC7A11 forms a cystine/glutamate antiporter with the heavy chain subunit of SLC3A2 [28]. SLC7A11 is highly specific for cystine and glutamate and plays a role in the uptake of cystine and release of glutamate. It has been demonstrated that this process can promote the synthesis of glutathione (GSH), thereby protecting cells from damage caused by oxidative stress and preventing cell death due to lipid peroxidation [29].

It has been demonstrated that the expression of specific miRNAs is significantly altered in certain disease states. Consequently, these miRNAs are regarded as potential biomarkers or therapeutic targets. A number of miRNA mimics or oligonucleotide inhibitors currently undergoing Phase I studies are listed on the Clinicaltrials.gov website, including MRG-106 or MRG-201 [30]. MRG-106 is a miR-155 oligonucleotide inhibitor (trade name Cobomarsen), which is used for the treatment of cutaneous T-cell lymphoma and is in Phase I clinical trials [31]. MRG-201 is a miR-29 mimetic, which is currently used in a variety of pathological fibrotic conditions and systemic sclerosis and is now in Phase I clinical trials. Small nucleic acid drugs represented by miRNAs also have relevant preclinical trials in the fields of oncology, rare diseases, viral hepatitis, and cardiovascular diseases, suggesting their wide application in the treatment of human diseases. Recent studies have found that miRNAs have the ability to promote osteogenic differentiation, but miRNAs have the problems of short half-life and easy degradation [32]. This study revealed that miR-181a directly targets TP53 mRNA, thereby inhibiting the TP53/SLC7A11-mediated ferroptosis pathway and alleviating ferroptosis-mediated cell death. The results of our study suggest that miR-181a may represent a novel therapeutic approach for bone loss.

## Data Availability

The original contributions presented in the study are included in the article/Supplementary Material, further inquiries can be directed to the corresponding authors.

## Supplementary Materials

The following supporting information can be downloaded at: https://www.mdpi.com/article/10.3390/jfb15120385/s1, Table S1: The infrared absorption bands positions of the samples; Figure S1: Flow cytometry was used to validate the surface marker genes of human MSCs; Figure S2: The H&E staining was performed to visualize the cytotoxicity of nHAC in different organs of rats; Figure S3: The immunohistochemical (IHC) staining was performed to visualize the expression of Osteopontin (OPN) in the rat tissues at 4 weeks. Scale bar: 100 μm.
