# Supplementary material for: miR-181a/MSC-Loaded Nano-Hydroxyapatite/Collagen Accelerated Bone Defect Repair in Rats by Targeting Ferroptosis Pathway
Source: J Funct Biomater. 2024 Dec 20;15(12):385. doi: 10.3390/jfb15120385 (PMC11677460; doi:10.3390/jfb15120385)
Supplement: Supplementary file 1 [file jfb-15-00385-s001.zip › jfb-3271121-supplementary.pdf]

Supplementary Table S1 The infrared absorption bands positions of the samples

Infrared vibration modes	FTIR positions (cm^{-1})	Functional groups
P-O bending	462*、472	PO_4
P-O bending	561、575*、601	PO_4
C-O bending	871 (Type A)、879 (Type B)	AB-type CO_3
P-O stretching	962、1026、1044、1066、1092*	PO_4
C-N stretching/ N-H bending	1231、1240、1250	Amide III
C-H bending	1335	CH_2
C-H bending	1381	CH_3
C-O stretching	1408 (Type B)、1450 (Type AB)、1558* (Type A)	AB-type CO_3
C-O stretching	1420、1460	B-type CO_3
N-H bending/ C-N stretching	1535	Amide II
C=O stretching	1634	Amide I
C-H stretching	2899、2969	CH_2
C-N stretching/ C-H stretching	2990	Amide B
N-H stretching	3298	Amide A
O-H stretching	3673	H_2O

*It indicates a shoulder peak around the characteristic peaks.

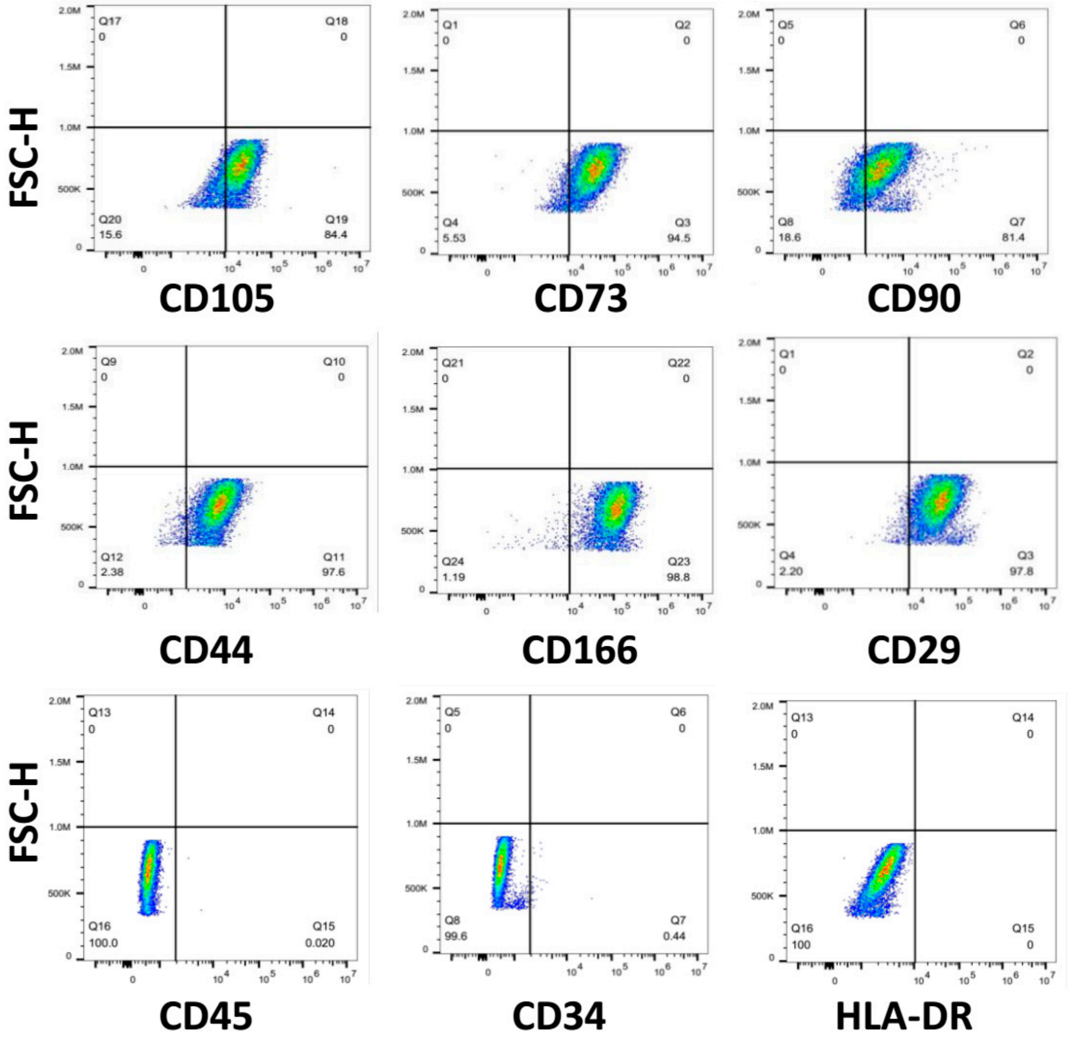

Supplementary Figure S1. Flow cytometry was used to validate the surface marker genes of human MSCs.

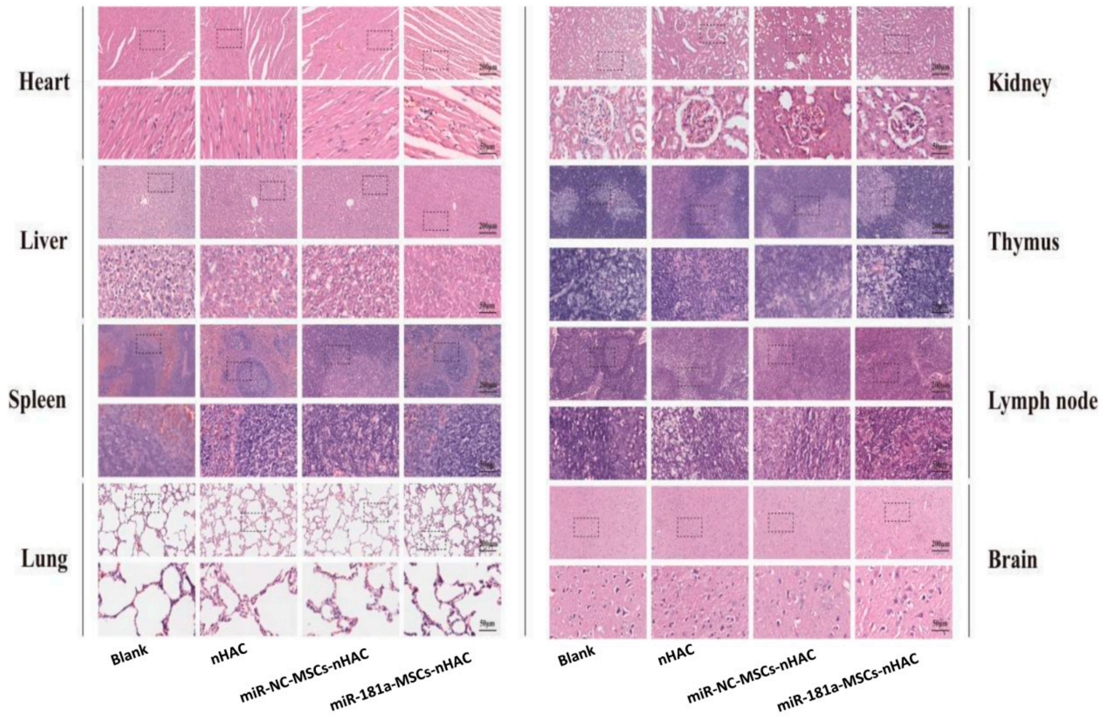

Supplementary Figure S2. The H&E staining was performed to visualize the cytotoxicity of nHAC in different organs of rats.

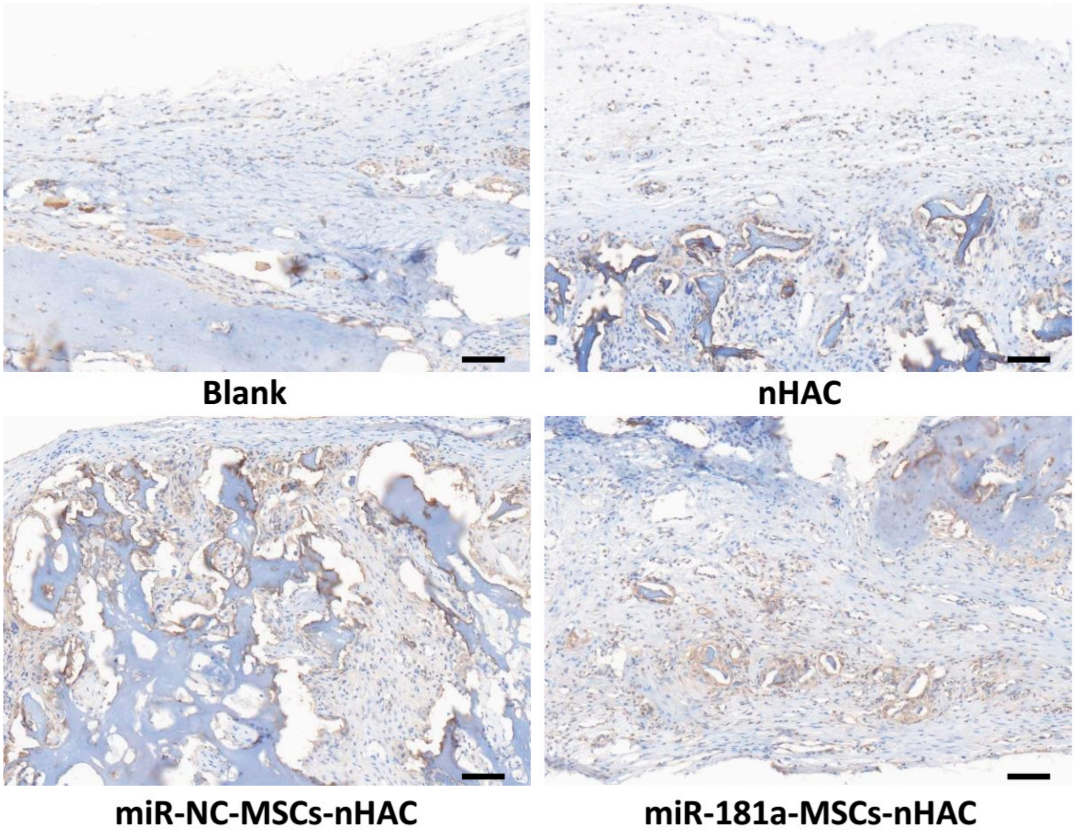

Supplementary Figure S3. The immunohistochemical (IHC) staining was performed to visualize the expression of Osteopontin (OPN) in the rat tissues at 4 weeks. Scale bar: 100μm.
